# Neurodegeneration and Vascular Burden on Cognition After Midlife: A Plasma and Neuroimaging Biomarker Study

**DOI:** 10.3389/fnhum.2021.735063

**Published:** 2021-12-14

**Authors:** Kuo-Lun Huang, Ing-Tsung Hsiao, Ting-Yu Chang, Shieh-Yueh Yang, Yeu-Jhy Chang, Hsiu-Chuan Wu, Chi-Hung Liu, Yi-Ming Wu, Kun-Ju Lin, Meng-Yang Ho, Tsong-Hai Lee

**Affiliations:** ^1^Department of Neurology, Linkou Chang Gung Memorial Hospital, and College of Medicine, Chang Gung University, Taoyuan, Taiwan; ^2^Department of Nuclear Medicine and Molecular Imaging Center, Linkou Chang Gung Memorial Hospital, Taoyuan, Taiwan; ^3^Healthy Aging Research Center and Department of Medical Imaging and Radiological Sciences, College of Medicine, Chang Gung University, Taoyuan, Taiwan; ^4^MagQu Co., Ltd., New Taipei City, Taiwan; ^5^Department of Radiology, Linkou Chang Gung Memorial Hospital, Taoyuan, Taiwan; ^6^Graduate Institute of Behavioral Sciences, Chang Gung University, Taoyuan, Taiwan

**Keywords:** cognition, amyloid plaque, PET, post-stroke cognitive impairment, plasma biomarker, tau protein

## Abstract

**Background and Objectives**: Neurodegeneration and vascular burden are the two most common causes of post-stroke cognitive impairment. However, the interrelationship between the plasma beta-amyloid (Aβ) and tau protein, cortical atrophy and brain amyloid accumulation on PET imaging in stroke patients is undetermined. We aimed to explore: (1) the relationships of cortical thickness and amyloid burden on PET with plasma Aβ40, Aβ42, tau protein and their composite scores in stroke patients; and (2) the associations of post-stroke cognitive presentations with these plasma and neuroimaging biomarkers.

**Methods**: The prospective project recruited first-ever ischemic stroke patients around 3 months after stroke onset. The plasma Aβ40, Aβ42, and total tau protein were measured with the immunomagnetic reduction method. Cortical thickness was evaluated on MRI, and cortical amyloid plaque deposition was evaluated by ^18^F-florbetapir PET. Cognition was evaluated with Mini-Mental State Examination (MMSE), Geriatric Depression Scale (GDS), Dementia Rating Scale-2 (DRS-2).

**Results**: The study recruited 24 stroke patients and 13 normal controls. The plasma tau and tau*Aβ42 levels were correlated with mean cortical thickness after age adjustment. The Aβ42/Aβ40 ratio was correlated with global cortical ^18^F-florbetapir uptake value. The DRS-2 and GDS scores were associated with mean cortical thickness and plasma biomarkers, including Aβ42/Aβ40, tau, tau*Aβ42, tau/Aβ42, and tau/Aβ40 levels, in stroke patients.

**Conclusion**: Plasma Aβ, tau, and their composite scores were associated with cognitive performance 3 months after stroke, and these plasma biomarkers were correlated with corresponding imaging biomarkers of neurodegeneration. Further longitudinal studies with a larger sample size are warranted to replicate the study results.

## Introduction

Neurodegeneration and vascular pathology are the two most common causes of cognitive impairment in the elderly. Post-stroke cognitive impairment (PSCI) is a special condition of vascular cognitive impairment (VCI) and refers to the presence of cognitive impairment after stroke. PSCI is noted in about one-third of stroke survivors, which usually manifest 3–6 months after stroke occurrence (Pendlebury and Rothwell, 2009; Gottesman and Hillis, 2010). The occurrence of PSCI can be attributed to vascular injury, pre-existing neurodegenerative substrates, and a combination of both (Mijajlović et al., 2017). Besides, stroke patients with co-existing beta-amyloid (Aβ) plaque tend to present steeper cognitive declines in longitudinal follow-up (Liu et al., 2015).

Regarding the *in vivo* detection of neurodegenerative pathology in stroke patients, positron emission tomography (PET) imaging, cerebrospinal fluid (CSF) and blood examination have been developed in the past decades. PET imaging can yield an excellent visual resolution of the topographical distribution of Aβ pathology and tau protein, but it is expensive with limited availability and requires multiple scanning sessions if different pathological substrates are to be detected. The concentrations of Aβ protein and tau protein in CSF have been shown highly correlated with brain pathological changes and clinical presentations, but the invasive nature of lumbar puncture limits its clinical application. Compared to PET and CSF studies, blood biomarkers of neurodegeneration have the advantage of more convenient accessibility with no risk for radiation exposure and invasive procedures (Blennow, 2017). Detection of plasma neurodegeneration markers, such as Aβ40, Aβ42, and tau protein, has been shown feasible and reliable by the immunomagnetic reduction (IMR) technique (Yang et al., 2011). Patients with Alzheimer’s disease (AD) had lower plasma Aβ40 and higher plasma Aβ42 and tau protein levels than patients with mild cognitive impairment (MCI) and healthy controls (Chiu et al., 2013, 2014). Regarding the reliability of plasma neurodegenerative biomarkers, the CSF and plasma Aβ42 were shown correlated in a recent AD study (Teunissen et al., 2018). Moreover, plasma Aβ40 and Aβ42 levels were significantly correlated with amyloid accumulation on Pittsburgh compound B (PiB) PET (Tzen et al., 2014).

Detection of fluid biomarkers of amyloid plaque and tau protein has been applied in stroke patients, and their levels would vary according to sampling time point, vascular lesion characteristics, and coexisting neurodegeneration states (Hesse et al., 2000; Zhang et al., 2010; Bielewicz et al., 2011; Skillback et al., 2015). Previous studies have shown that there is no significant change in amyloid plaque accumulation after acute stroke (Hesse et al., 2000; Sahathevan et al., 2016). On the other hand, tau protein level has an abrupt increase within 5–10 days after acute stroke, and then gradually decreases to a stable level in the following 3 months after stroke (Hesse et al., 2000; Kaerst et al., 2013). Therefore, plasma amyloid peptide and tau protein levels measured 3 months after stroke would be within a relatively stable condition and may represent the overall neurodegenerative condition in stroke patients.

Although plasma Aβ42 and tau protein levels have been recently reported to have significant associations with VCI presentations (Tang et al., 2018; Chi et al., 2019), there is limited literature on the relationship between the imaging and blood biomarkers of neurodegeneration in the context of PSCI. In this study, we aimed to explore: (1) the associations of plasma Aβ40, Aβ42, and tau protein levels with cognitive presentations around 3 months after first-ever ischemic stroke; and (2) the relationships of plasma Aβ40, Aβ42, and tau protein with the relevant imaging markers, such as cortical atrophy on MRI and amyloid burden on PET imaging.

## Methods

### Participants

We conducted a prospective, cross-sectional study to screen patients with recent first-ever ischemic stroke (around 3 months after onset) from the Department of Neurology and Stroke Center at Linkou Chang Gung Memorial Hospital, Taiwan, as previously described (Huang et al., 2018b). These stroke patients were recruited based on the following criteria: (1) a diagnosis of acute ischemic stroke confirmed on magnetic resonance imaging (MRI) at stroke onset; (2) education years at least 6 years; (3) no history of old stroke, dementia, tauopathy diseases, substantial traumatic brain injury or epilepsy before the index stroke; (4) without recurrent stroke occurring between the index stroke and the study screening procedure; and (5) without persistent moderate to severe dysphasia, which was defined as a score of >1 point in the language score of the National Institutes of Health Stroke Scale (NIHSS; Srikanth et al., 2006). The NIHSS scores were recorded at stroke onset and 3 months after stroke. In addition, age- and education-matched elderly normal controls were also recruited, and they had: (1) education at least 6 years; (2) no subjective cognitive complaint; (3) no major neurological and psychiatric disease; and (4) the sum of Clinical Dementia Rating sub-scores was 0.

The study protocol and procedure for obtaining informed consent were compliant with the Helsinki Declaration and were approved by the Institutional Review Board of Chang Gung Memorial Hospital (IRB No. 201601092B0, 201601675A0, 103-7584A). All participants provided written informed consent.

### Cognitive Assessment

Cognitive assessment was administered on all participants around 3 months after the occurrence of the index stroke. The assessment entailed the Mini-Mental State Examination (MMSE), the Clinical Dementia Rating (CDR) and the Dementia Rating Scale-2 (DRS-2). The subtests of the DRS-2, including attention, initiation and perseveration (IP), conceptualization, memory and construction, were applied to evaluate domain-specific cognitive function. The 15-item Geriatric Depression Scale (GDS) was used to evaluate mood condition. All of these tests have been used in our previous studies (Huang et al., 2017, 2018a).

### Blood Sample Collection and Preparation

Every participant was asked to provide a 10-ml non-fasting venous blood sample (K3 EDTA, lavender-top tube). The blood samples were centrifuged (3,000 *g* for 20 min) within 1 h of collection, and plasma was aliquoted into cryotubes and stored at −80°C before measurement. The laboratory staff were blinded to the demographic, clinical and imaging data of each participant.

### IMR Measurements

Measurements of plasma Aβ40, Aβ42 and total tau protein with immunomagnetic reduction (IMR) have been reported in our previous study (Lin et al., 2019). In brief, the reagents used to determine plasma Aβ40, Aβ42, and tau protein levels in this study consisted of dextran-coated Fe_3_O_4_ nanoparticles functionalized with antibodies. Immunomagnetic reduction assays were the method used to probe the associations of plasma magnetic nanoparticles with Aβ40, Aβ42, and tau protein reagents. This technique mainly detected the percentage reduction in an alternating current that reflects the magnetic susceptibility (*X*ac) of a reagent due to the interactions of functionalized magnetic nanoparticles and target proteins. The percentage reductions of immunomagnetic signals were then converted to target protein concentrations using the standard curves of the respective analytes.

### Stroke Volume and Brain Atrophy Evaluation

Brain CT and MRI were performed at stroke onset to assess acute stroke lesions. The MRI scanning protocol included fluid-attenuated inversion recovery (FLAIR), diffusion-weighted imaging (DWI), and T1-weighted (T1W) sequences. The stroke volume was delineated on the DWI maps using the PMOD software (version 3.7; PMOD Technologies Ltd., Zurich, Switzerland). We normalized stroke lesion volume according to the head size, which was measured using the FreeSurfer software (version 6.0.0).

Brain atrophy was evaluated based on the follow-up brain MRI scans performed around 3 months after stroke onset. Axial three-dimensional T1W-MPRAGE (Magnetization Prepared Rapid Gradient Echo) and FLAIR sequences were acquired on a Siemens 3T MRI system as previously described (Huang et al., 2018b). We measured the cortical thickness on the T1W-MPRAGE images using the FreeSurfer software (Becker et al., 2011). We evaluated hippocampal atrophy using the Schelten medial temporal lobe atrophy (MTA) score (Scheltens and van de Pol, 2012; Huang K. L. et al., 2019).

### Amyloid PET Image Acquisition and Analysis

An ^18^F-florbetapir PET scan was performed using Biograph mMR PET/magnetic resonance scanner (Siemens Medical Solutions, Malvern, PA, USA) about 3 months after stroke onset. A 10-min PET scan of ^18^F-florbetapir was acquired at 50 min post-injection of 384 ± 13 MBq. ^18^F-florbetapir PET images were reconstructed using point-spread function reconstruction with two iterations and 21 subsets, as well as MR-based attenuation correction and scatter and random corrections. The final reconstructed ^18^F-florbetapir PET images were of 344 × 344 × 127 matrix size (0.834 × 0.834 × 1.2 mm voxel size).

PET data were motion-corrected, and then spatially normalized into MNI space using MR-based spatial normalization. Image processing was performed using PMOD software (version 3.7; PMOD Technologies Ltd, Zurich, Switzerland) by previously reported protocols (Huang C.-C. et al., 2019). Then, the SUVR (standardized uptake value ratio) image was calculated by using the cerebellar gray matter as the reference region. Amyloid plaque positivity was visually evaluated on ^18^F-florbetapir PET images (Johnson et al., 2013).

### Study Procedures and Statistical Analyses

Cognitive performance, plasma neurodegenerative biomarkers, mean cortical thickness on brain MRI, and ^18^F-florbetapir SUVR as amyloid burden were measured around 3 months after acute stroke. Firstly, we evaluated the influence of plasma neurodegenerative markers, such as Aβ40, Aβ42, and tau protein, on PSCI performance. Secondly, we evaluated the associations of plasma neurodegenerative markers with the corresponding neuroimaging markers, including mean cortical thickness and ^18^F-florbetapir SUVR.

For descriptive statistics, we performed the two-sample t-test, chi-square test, and Fisher’s exact test for group comparisons. Further, we performed Pearson’s correlation analyses to investigate the correlations among the mean cortical thickness, ^18^F-florbetapir SUVR, and plasma biomarker values. Moreover, we analyzed the partial correlations of cognitive performance with plasma biomarker and other relevant factors after adjusting for age and education.

## Results

The study recruited 24 patients with first-ever ischemic stroke and 13 age- and education-matched normal controls. Stroke patients had higher proportions of hypertension and dyslipidemia than those of the controls. There were no differences between the two groups in mean cortical thickness, plasma levels of Aβ40, Aβ42, tau, or the composite scores for Aβ42/Aβ40, tau/Aβ40, tau*Aβ42, and tau*Aβ42/Aβ40 (Table 1). The median days of blood sampling, neurocognition assessment, and neuroimaging evaluation were 96 (86–105, interquartile range), 98 (88–110), and 100 (87–118) days after stroke occurrence for stroke patients, and these intervals were highly correlated (*r* = 0.48–0.68, *p* values < 0.03). These evaluations were performed within 15 days for the controls.

**Table 1:** Comparisons of demographic data and plasma Aβ40, Aβ42, tau protein and their composite scores between normal controls and patients with ischemic stroke.

	NC, *n* = 13	Stroke, *n* = 24	Effect size	*p* value
**Categorical variables**	n (%)	n (%)	Odds Ratio	
Male	7 (54)	20 (83)	4.3 (0.9–19.8)	0.12
APOE4 carrier	0 (0)	3 (13)	NA	0.54
Hypertension	6 (46)	21 (88)	8.2 (1.6–41.6)	0.02
Diabetes mellitus	3 (23)	7 (29)	1.4 (0.3–6.5)	1.00
Dyslipidemia	5 (38)	18 (75)	4.8 (1.1–20.5)	0.04
Gout	1 (8)	5 (21)	3.2 (0.3–30.4)	0.39
**Continuous variables**	Mean (SD)	Mean (SD)	Cohen’s d	
Age, y	64.8 (6.3)	62.0 (8.5)	0.37	0.29
Education, y	10.5 (3.5)	10.5 (3.3)	0.01	0.97
Mean cortical thickness, mm	2.40 (0.06)	2.41 (0.07)	0.15	0.66
MTA score	0.9 (1.0)	0.7 (0.9)	0.03	0.58
Aβ40, pg/ml	53.2 (3.2)	53.1 (6.8)	0.01	0.98
Aβ42, pg/ml	16.5 (0.5)	16.3 (1.4)	0.14	0.61
Tau, pg/ml	21.4 (2.5)	19.7 (4.5)	0.42	0.16
Aβ42/Aβ40	0.31 (0.03)	0.31 (0.07)	0.06	0.82
Tau/Aβ40	0.40 (0.06)	0.39 (0.14)	0.15	0.59
Tau/Aβ42	1.30 (0.12)	1.20 (0.20)	0.54	0.08
Tau*Aβ42	353.5 (50)	326.7 (95.1)	0.32	0.27
Tau*Aβ42/Aβ40	6.70 (1.17)	6.45 (2.79)	0.10	0.71
MMSE	26.9 (1.7)	26.7 (2.1)	0.13	0.71
GDS	1.3 (1.7)	2.0 (2.5)	0.31	0.38
DRS-2 total	131.9 (7.2)	129.4 (10.1)	0.28	0.43
DRS-2 attention	35.3 (1.4)	36.0 (1.0)	0.64	0.07
DRS-2 IP	31.8 (3.9)	29.5 (5.6)	0.45	0.20
DRS-2 Conceptualization	36.2 (2.2)	35.4 (4.0)	0.25	0.41
DRS-2 Memory	22.6 (1.9)	22.4 (2.1)	0.10	0.78
DRS-2 Construction	5.9 (0.3)	6.0 (0.0)	0.47	0.34
CDR SOB	0 (0)	0.3 (0.4)	0.79	0.01
Stroke volume, 10–^6^	NA	3.92 (5.36)	NA	NA
NIHSS score at onset	NA	2.6 (1.4)	NA	NA
NIHSS score at 3M	NA	1.7 (1.1)	NA	NA

*NC, normal controls; APOE, apolipoprotein E; MTA, medial temporal atrophy; MMSE, Mini-Mental State Examination; GDS, Geriatric Depression Scale; DRS-2, Dementia Rating Scale-2; IP, Initiation and Perseveration; CDR SOB, sum of boxes of Clinical Dementia Rating Scale; NIHSS, National Institutes of Health Stroke Scale; NA, not applicable*.

**Figure 1:**
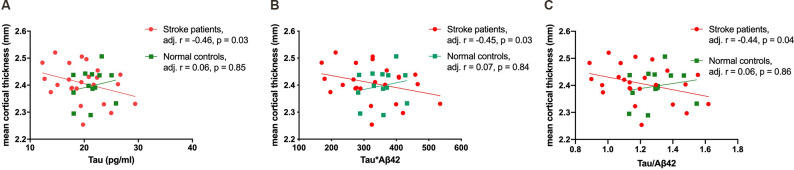
The correlations of mean cortical thickness with plasma tau **(A)**, tau*Aβ42 **(B)**, and tau/Aβ42 **(C)** in stroke patients and normal controls.

Age was negatively correlated with mean cortical thickness in all participants (*r* = −0.35, *p* value = 0.04), and plasma tau protein level and tau-related composite scores were correlated with mean cortical thickness after age adjustment. Subgroup analyses were then performed to evaluate associations between plasma biomarkers and mean cortical thickness in stroke patients and normal controls, respectively. In stroke patients, plasma tau, tau*Aβ42, and tau/Aβ42 levels were correlated with mean cortical thickness, but not with stroke volume (Figure 1). However, such correlations were not observed in normal controls (Table 2).

**Table 2:** Partial correlations of mean cortical thickness with plasma Aβ40, Aβ42, and tau protein and their composite scores after adjustment for age.

	Aβ42	Aβ40	Aβ42/Aβ40	Tau	Tau*Aβ42	Tau/Aβ42	Tau/Aβ40	Tau*Aβ42/Aβ40
All subjects	−0.31	0.25	−0.30	−0.34*	−0.34*	−0.31	−0.33*	−0.34*
Stroke	−0.39	0.27	−0.34	−0.46*	−0.45*	−0.44*	−0.41	0.41
NC	0.06	0.18	−0.09	0.06	0.07	0.06	−0.01	0.01

*P < 0.05.

**Figure 2:**
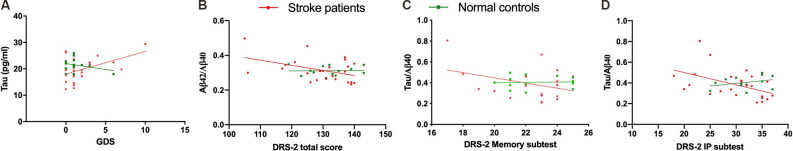
The correlations among cognitive results and plasma biomarkers. Geriatric Depression Score (GDS) vs. tau **(A)**. Dementia Rating Scale-2 (DRS-2) total score vs. Aβ42/Aβ40 ratio **(B)**. DRS-2 memory subtest score vs. tau/Aβ40 **(C)**. DRS-2 initiation and perseveration (DRS IP) subtest score vs. tau/Aβ40 **(D)**.

In all participants, plasma levels of Aβ40, Aβ42/Aβ40, tau/Aβ40, and tau*Aβ42/Aβ40 were all significantly correlated with the GDS and DRS-2 total scores, respectively, and remained significant after controlling for age and education (Table 3). Similarly, in stroke patients alone, the plasma Aβ42/Aβ40, tau, tau/Aβ42, tau/Aβ40, and tau*Aβ42/Aβ40 levels were significantly correlated with the GDS score, DRS-2 total score, and DRS-2 I/P subtest score after age and education adjustment (Figure 2). The correlations of stroke volume with the NIHSS, MMSE, GDS and DRS-2 total scores were not significant.

**Table 3:** The correlations of cognitive results with cortical thickness, stroke volume, plasma Aβ42, Aβ40, tau, and their composite scores.

Cognitive tests	Groups	Cortical thickness	Aβ42	Aβ40	Aβ42/Aβ40	Tau	Tau*Aβ42	Tau/Aβ42	Tau/Aβ40	Tau*Aβ42/Aβ40	Stroke volume
MMSE	All	0.32	0.03	0.23	-0.15	-0.08	-0.05	-0.12	-0.15	-0.12	NA
	IS	0.37	0.02	0.29	-0.19	-0.04	-0.02	-0.07	-0.16	-0.12	-0.09
	NC	0.20	0.10	-0.03	0.09	-0.30	-0.23	-0.40	-0.20	-0.14	NA
GDS	All	-0.37*	0.23	-0.35*^†^	0.38*^†^	0.31^†^	0.32^†^	0.29	0.41*^†^	0.40*^†^	NA
	IS	-0.41*	0.31	-0.41*	0.44*^†^	0.48*^†^	0.46*^†^	0.48*^†^	0.52*^†^	0.51*^†^	-0.16
	NC	-0.33	-0.22	-0.04	-0.06	-0.29	-0.28	-0.30	-0.22	-0.21	NA
DRS-2 Total	All	0.33*	-0.18	0.39*^†^	-0.38*^†^	-0.27*^†^	-0.26^†^	-0.28	-0.39*^†^	-0.37*^†^	NA
	IS	0.41*	-0.23	0.47*	-0.44*^†^	-0.41*^†^	-0.38^†^	-0.44*^†^	-0.50*^†‡^	-0.46*^†‡^	-0.13
	NC	0.17	0.10	0.01	0.02	0.16	0.16	0.16	0.12	0.12	NA
DRS-2 Attention	All	0.32	-0.09	0.26	-0.19	-0.14	-0.13	-0.14	-0.19	-0.18	NA
	IS	0.23	-0.04	0.25	-0.20	-0.14	-0.12	-0.16	-0.22	-0.20	-0.31
	NC	0.44	-0.24	0.45	-0.40	0.03	-0.03	0.10	-0.16	-0.17	NA
DRS-2 IP	All	0.27	-0.23^†^	0.35*^†^	-0.35*^†^	-0.26	-0.26^†^	-0.25	-0.34*^†^	-0.32*^†^	NA
	IS	0.37	-0.32^†‡^	0.44*^†‡^	-0.43*^†‡^	-0.46*^†‡^	-0.43*^†‡^	-0.47*^†‡^	-0.48*^†‡^	-0.45*^†‡^	-0.21
	NC	0.07	0.27	-0.15	0.19	0.34	0.34	0.33	0.33	0.32	NA
DRS-2 Conceptualization	All	0.21	-0.09	0.25	-0.27	-0.16	-0.15	-0.16	-0.28	-0.27	NA
	IS	0.23	-0.11	0.29	-0.30	-0.21	-0.19	-0.22	-0.32	-0.30	0.22
	NC	0.20	-0.04	0.03	-0.06	-0.05	-0.05	-0.05	-0.07	-0.07	NA
DRS-2 Memory	All	0.27	0.00	0.29^†^	-0.23^†^	-0.19^†^	-0.16	-0.24^†^	-0.29^†^	-0.26^†^	NA
	IS	0.41*	-0.02	0.38^†^	-0.30	-0.27^†^	-0.22	-0.33^†^	-0.38^†^	-0.34^†^	-0.33
	NC	-0.04	0.10	-0.06	0.09	0.02	0.04	-0.01	0.04	0.04	NA
DRS-2 Construction	All	-0.09	-0.10	0.08	-0.07	-0.20	-0.19	-0.22	-0.15	-0.14	NA
	IS	NA	NA	NA	NA	NA	NA	NA	NA	NA	NA
	NC	-0.19	-0.35	0.25	-0.30	-0.44	-0.45	0.42	-0.46	-0.45	NA
NIHSS	All	0.01	-0.08	-0.09	0.07	-0.05	-0.05	-0.05	0.05	0.04	NA
	IS	-0.07	-0.05	-0.13	0.06	0.14	0.09	0.20	0.14	0.11	-0.11
	NC	NA	NA	NA	NA	NA	NA	NA	NA	NA	NA
CDR SOB	All	-0.31	0.06	-0.17	0.13	0.03	0.04	0.02	0.09	0.09	NA
	IS	-0.44*	0.10	-0.19	0.14	0.13	0.11	0.14	0.14	0.12	0.00
	NC	NA	NA	NA	NA	NA	NA	NA	NA	NA	NA

*P < 0.05; ^†^*P* < 0.05 after age and education adjustment; ^‡^*P* < 0.05 after age, education and GDS adjustment; NA, not applicable; MMSE, Mini-Mental State Examination; GDS, Geriatric Depression Scale; DRS-2, Dementia Rating Scale-2; IP, Initiation and Perseveration; NIHSS, National Institutes of Health Stroke Scale; CDR SOB, the sum of boxes of Clinical Dementia Rating Scale; IS, ischemic stroke; NC, normal controls.

**Figure 3:**
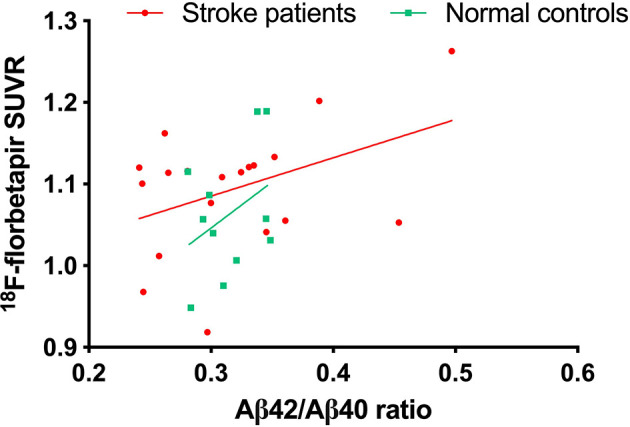
The correlation between plasma Aβ42/Aβ40 ratio and cortical amyloid accumulation burden on ^18^F-florbetapir PET.

An ^18^F-florbetapir PET scanning was done in 19 of 24 stroke patients and 11 of 13 controls. There were no differences in age, education, or cognitive test scores between participants with and without ^18^F-florbetapir PET scanning. The ^18^F-florbetapir PET imaging was visually negative for amyloid plaque in all participants. The ^18^F-florbetapir SUVR was positively and moderately correlated with Aβ42/Aβ40 (*r* = 0.42, *p* value = 0.07) in stroke patients (Figure 3), but was not significantly correlated with any cognitive test scores in either group.

## Discussion

PSCI is not a single disease entity; rather, it describes an unspecified cognitive decline that usually occurs 3–6 months after stroke onset (Tuladhar and de Leeuw, 2010). In this study, we checked plasma Aβ42, Aβ40, and tau protein levels as well as structural MRI and amyloid PET scanning around 3 months after the occurrence of first-ever ischemic stroke. We found that plasma tau protein level and tau-related composite scores were correlated with mean cortical thickness. In addition, the Aβ42/Aβ40 ratio was moderately correlated with ^18^F-florbetapir PET SUVR. Furthermore, plasma Aβ42, Aβ40, tau protein and their composite scores were correlated with cognitive performance 3 months after stroke. All these findings suggest plasma biomarkers of Aβ42, Aβ40, and tau protein may be associated with the development of PSCI.

Amyloid plaque is a pathognomonic marker of Alzheimer’s disease and could be present for decades before the manifestations of cognitive impairment (Jack et al., 2013). Although vascular lesions and Aβ pathology frequently coexist in stroke patients, previous CSF or amyloid PET studies have shown that stroke itself would not induce amyloid plaque accumulation (Hesse et al., 2000; Sahathevan et al., 2016). Nonetheless, the presence of co-existing amyloid plaque has been implicated as a risk factor for PSCI development (Thiel et al., 2014; Skillback et al., 2015). Therefore, detection of both vascular injury and neurodegeneration pathology would be helpful to disentangle the complex etiologies of PSCI. Our study found that plasma Aβ proteins were correlated with PSCI performance. Of note, the Aβ42/Aβ40 ratio was more sensitive to PSCI than Aβ42 or Aβ40 alone in our study. It has been proposed that the Aβ42/Aβ40 ratio could compensate for general inter-individual variations than Aβ42 or Aβ40 alone (Hansson et al., 2019), and the Aβ42/Aβ40 ratio in either CSF and blood studies had adequate accuracy and reliability to differentiate AD patients from controls (Chiu et al., 2012; Hansson et al., 2019).

The correlations of VCI with plasma Aβ42, Aβ40, and tau protein have been recently reported (Tang et al., 2018; Chi et al., 2019). Tang et al. (2018) found elevated plasma Aβ42 was associated with worse cognitive performance in stroke patients. However, the intervals from stroke occurrence to cognitive evaluation ranged from acute stroke stage to more than 10 years after stroke in their study, and disparity in evaluation intervals may limit the ability to elucidate the temporal relationship between vascular and neurodegenerative contributions to the PSCI development. In our study, the plasma biomarker collection, cognitive evaluation and neuroimaging studies were performed around 3 months after stroke, and we found the plasma Aβ42/Aβ40 ratio and tau-related composite scores were correlated with PSCI presentations. This finding implies the potential role of these markers in signifying the development of PSCI. On the other hand, we found that stroke volume was not correlated with PSCI severity in this study. This could be partly related to the relatively minor stroke severity in our cases, whose NIHSS scores were 2.6 ± 1.4 points at stroke onset and 1.7 ± 1.1 points 3 months after stroke, respectively.

Both plasma and CSF Aβ42/Aβ40 ratios have been reported as surrogate biomarkers of cortical amyloid plaque deposition on PET (Fandos et al., 2017; Alcolea et al., 2019). Although the ^18^F-florbetapir PET results were visually rated negative for Aβ pathology in our stroke patients, the plasma Aβ42/Aβ40 ratio had a moderate correlation with global ^18^F-florbetapir SUVR. In agreement with the previous studies, our results showed that the plasma Aβ42/Aβ40 ratio was correlated with cognitive performance as well as the ^18^F-florbetapir SUVR even under the condition of low Aβ burden on PET imaging.

Tau is a microtubule-associated protein involved in stabilizing the axonal cytoskeleton and is deemed as a potential marker of axonal injury (Seco et al., 2012). Previous CSF and serum studies have shown that tau protein level has an abrupt elevation in the acute ischemic stroke stage, with a peak increase within 5–10 days after stroke onset, followed by a gradual normalization after 3 months. Therefore, the interval from stroke onset to blood collection may have an influence on the plasma tau protein level. In our study, plasma tau protein was collected around 3 months after stroke, and intervals from stroke onset to plasma collection were not correlated with plasma tau level or its composite scores (data not shown). Furthermore, the tau protein level in the acute stage is correlated with stroke volume, implying the role in direct neuronal injury severity (Hesse et al., 2000; Bitsch et al., 2002; Bielewicz et al., 2011; Kaerst et al., 2013). However, the relationship between acute tau protein level and post-stroke cognitive performance has not been well investigated. In a recent study, a single measurement of plasma tau protein which was done within 1 week after stroke onset has not revealed an association with cognitive performance 3 months or 1 year after stroke. Since a steep elevation of tau protein level may occur within days after stroke onset, multiple and intensive sampling of tau protein levels in the acute stroke stage should be more reliable when investigating its influence on long-term neurological and cognitive deficits in future studies.

On the other hand, since the plasma tau protein was detected around 3 months after stroke in our study, its concentration might be more related to either pre-existing or stroke-related neurodegeneration, rather than the direct neuronal injury effect (Chen and Jiang, 2019). Furthermore, we found plasma tau protein level and its composite scores were associated with mean cortical thickness and cognitive performance in stroke patients. Similar findings were also noted in previous studies. Tau protein level is associated with brain atrophy severity in both stroke patients and normal controls (Ihle-Hansen et al., 2017; Harrison et al., 2021). However, stroke patients have a greater brain atrophy rate than normal controls (Brodtmann et al., 2020), and the correlation between CSF tau protein level and brain atrophy severity is still significant 1 year after stroke, suggesting stroke may enhance or trigger tau-linked neurodegeneration with loss of neurons (Ihle-Hansen et al., 2017).

Various combinations of plasma Aβ42, Aβ40, and tau levels have shown better correlations with cognition than single biomarkers in patients with either AD or VCI (Lue et al., 2017; Chen et al., 2019; Chi et al., 2019). During the neurodegenerative process, Aβ tends to elevate during the MCI stage and reaches a plateau towards the demented stage, while tau protein increases as AD progresses. Therefore, the combination of these biomarkers could be synergistically representative of the neurodegeneration profile. For example, the product of plasma Aβ42 and tau has better accuracy for clinically diagnosed AD than either biomarker alone (Lue et al., 2017; Jiao et al., 2020). Moreover, the plasma tau/Aβ42 ratio showed a stronger association with brain tau accumulation than tau alone (Park et al., 2019). Indeed, the characteristic of an inverse relationship between plasma tau and Aβ40 during the neurodegeneration process (Fan et al., 2018) was also epitomized in our study that the tau/Aβ40 ratio had the strongest correlation with cognitive performance among the other plasma biomarkers and their composite scores.

PSCI development is subject to multiple factors, and the underlying mechanisms are not fully understood. In addition to AD-specific biomarkers, there is a considerable number of fluid and imaging biomarkers associated with vascular cognitive impairment. Previous studies have shown elevated CSF/blood albumin ratio, CSF matrix metalloproteinase (MMP) level, CSF neurofilament, and blood inflammatory cytokines and adhesion molecules are associated with worse cognitive performance, which could be attributed to disruption of blood-CSF/brain barriers and breakdown of white matter fibers and extracellular matrix (Wallin et al., 2017). Furthermore, neuroimaging measures, such as total gray matter volume, leukoaraiosis severity, stroke location, CSF volume, and neuroinflammatory presentations, have been implicated in PSCI development (Thiel et al., 2014; Molad et al., 2019; Huang et al., 2020). Therefore, multi-modality studies are required to investigate the relationships among fluid and imaging biomarkers and their composite influence on PSCI prognosis.

There were several limitations of this study that should be considered. First, the sample size was relatively small, and it should be cautious to generalize the study results. A larger sample size is required to validate the influence of plasma Aβ and tau protein on the PSCI development. Second, additional studies may be required to investigate the interactions among stroke location, stroke severity, and plasma AD biomarkers on the PSCI presentations. Since CSF biomarker analysis has been a more direct measure of the CNS condition than blood analysis, the correlations between CSF and blood measurement of neurodegeneration biomarkers, especially for tau protein, in stroke patients may be needed in future studies. Finally, this was a cross-sectional study, and further longitudinal study is deemed necessary to determine the long-term influence of plasma AD biomarkers on PSCI development.

## Conclusion

In this study, we found that plasma Aβ42/Aβ40 ratio was correlated with amyloid cortical deposition on ^18^F-florbetapir SUVR and the total tau protein value was correlated with mean cortical thickness 3 months after stroke. The plasma Aβ42/Aβ40 ratio, tau protein, and tau-related composite scores were correlated with cognitive performance. The relationship of plasma Aβ40, Aβ42, and tau protein with the long-term post-stroke structural and cognitive changes requires further studies in larger populations.

## Data Availability Statement

The datasets presented in this article are not readily available because the used consent does not allow for the public sharing of the data. Requests to access the datasets should be directed to thlee@adm.cgmh.org.tw.

## Ethics Statement

The studies involving human participants were reviewed and approved by The institutional review board at Linkou Chang Gung Memorial Hospital. The patients/participants provided their written informed consent to participate in this study.

## Author Contributions

K-LH wrote the initial draft, performed neurologic examinations, took part in the data collection and analysis, and scientific interpretation of data. K-JL conceptualized the study design, carried out PET imaging analysis, and wrote a portion of the draft. S-YY conceptualized the study design, took part in scientific interpretation of data, and critical review of the manuscript. T-YC, Y-JC, H-CW, and C-HL performed the data collection, neurologic examination, and scientific interpretation of data. I-TH and Y-MW performed the MRI analysis, took part in data interpretation, and critical review of the manuscript. M-YH conceptualized the study design, wrote a portion of the draft, conducted the cognitive evaluation, took part in data analysis, and critical review of the manuscript. T-HL conceptualized the study design, took part in critical review of the manuscript, and edited the manuscript for content. All authors contributed to the article and approved the submitted version.

## Conflict of Interest

S-YY is an employee and a shareholder of MagQu Co., Ltd. The remaining authors declare that the research was conducted in the absence of any commercial or financial relationships that could be construed as a potential conflict of interest.

## Publisher’s Note

All claims expressed in this article are solely those of the authors and do not necessarily represent those of their affiliated organizations, or those of the publisher, the editors and the reviewers. Any product that may be evaluated in this article, or claim that may be made by its manufacturer, is not guaranteed or endorsed by the publisher.
